# Megamimivirus double-stranded DNA linear genomes flanked by highly diverse terminal inverted repeats

**DOI:** 10.1128/jvi.00825-26

**Published:** 2026-09-09

**Authors:** Yucheng Xia, Lanlan Cai, Boyu Ding, Baiyu Su, Rui Zhang

**Affiliations:** 1 Earth, Ocean and Atmospheric Sciences Thrust, The Hong Kong University of Science and Technology (Guangzhou) 567841, Guangzhou, China; 2 Archaeal Biology Center, Synthetic Biology Research Center, Shenzhen Key Laboratory of Marine Microbiome Engineering, Key Laboratory of Marine Microbiome Engineering of Guangdong Higher Education Institutes, Institute for Advanced Study, Shenzhen University 620539, Shenzhen, China; Michigan State University, East Lansing, Michigan, USA

**Keywords:** *Megavirus*, *Mimivirus*, giant virus, terminal inverted repeat, transfer RNA

## Abstract

Giant viruses have fundamentally expanded our understanding of virology by challenging the conventional boundaries of both virion size and genome complexity. However, the scarcity of isolates has left many of their unique biological features unexplored. Here, we report the isolation and characterization of four new giant virus species belonging to the subfamily *Megamimivirinae*, sampled from distinct environments across China. Among these, Megavirus daqingense is the first giant virus isolated from an oil reservoir; it exhibits virion stability under high salinity, chloroform exposure, and elevated temperatures, suggesting fitness adaptations to subsurface conditions. Using a hybrid sequencing approach that integrates short- and long-read technologies, we assembled complete linear genomes for all four isolates, each flanked by long terminal inverted repeats (TIRs). Comparative genomic and synteny analyses identified 29 distinct TIRs from 46 megamimivirus genomes. Gene content within these TIRs was highly diverse, with no orthologous proteins conserved across all repeats. Furthermore, TIR genes experienced weaker purifying selection than those in non-TIR regions (i.e., the genomic regions excluding the TIRs), consistent with their role as drivers of genome plasticity. Notably, we discovered for the first time that identical tRNA genes are shared between TIRs and non-TIR regions of eukaryotic viruses. Collectively, our work provides insights into the structural and evolutionary complexity of megamimiviruses, revealing TIRs as reservoirs of genetic diversity and hotspots for gene transfer, thereby playing a pivotal role in shaping the dynamic architecture of giant virus genomes.

**IMPORTANCE:**

Terminal inverted repeats (TIRs) are critical structural elements at the termini of linear genomes essential for fundamental processes such as recombination, replication, and integration across diverse organisms. However, the inherent limitations of short-read sequencing technologies have left the complete structure, diversity, and evolutionary significance of long TIRs in giant viruses unexplored. In this study, we leverage hybrid sequencing and comparative genomic analyses to unveil the complexity of TIRs across the subfamily *Megamimivirinae*. We demonstrate that TIRs are dynamic genomic hotspots characterized by remarkable gene diversity and unexpected conservation of specific tRNA genes. These findings establish TIRs as key drivers of genome plasticity, serving as hotspots for horizontal gene transfer and genetic innovation. By resolving the long-hidden terminal structures of megamimivirus genomes, this work provides a foundational framework for understanding how TIRs shape the evolution of giant viruses and, more broadly, advances our understanding of genome architecture in large DNA viruses.

## INTRODUCTION

The discovery of *Acanthamoeba polyphaga* mimivirus (APMV, *Mimivirus bradfordmassiliense*) two decades ago fundamentally challenged the conventional definition of viruses, revealing a world of giant viruses with particle and genome sizes rivaling those of small bacteria (1, 2). Since then, the phylum *Nucleocytoviricota* has expanded to encompass an ever-growing diversity of giant viruses (3), such as *Tupanvirus* (4), *Yasminevirus* (5), *Bodo saltans* virus (*Theiavirus*) (6), and *Haptolina ericina* virus and *Prymnesium kappa* virus (*Tethysvirus*) (7), which infect a wide range of eukaryotic hosts, from amoebae to marine protists. Among these, APMV, a typical member of the subfamily *Megamimivirinae* (family *Mimiviridae*), stands out for its exceptionally large and complex genome, which features a conserved central region harboring core genes essential for replication and transcription, flanked by highly dynamic terminal regions (8–10).

Terminal inverted repeats (TIRs), also referred to as inverted terminal repeats, are a prevalent structural feature of many linear genomes across the tree of life (7, 11–21). These identical sequences, oriented in opposite directions at the two genome extremities, are critical for fundamental processes such as homologous recombination, replication stability, and genome packaging in viral systems, ranging from small viruses such as adenovirus (*Mastadenovirus*) (16) and adeno-associated virus (*Dependoparvovirus*) (17) to virophages such as *Sputnikvirus* and *Mavirus* (11, 12). In giant viruses, TIRs can reach tens of kilobases in length, accounting for a substantial fraction of the genome, as exemplified by the 23-kb TIRs in *Megavirus* (13, 20), the 10-kb TIRs in *Mollivirus* (15), and even the 96-kb TIRs in *Tethysvirus* (7). Despite their apparent prevalence and potential significance, the complete architecture, diversity, and functional significance of TIRs in giant viruses have remained elusive.

This obscurity stems primarily from the historical reliance on short-read next-generation sequencing (NGS) technologies, which are limited in their ability to assemble large, repetitive structures (22). Consequently, the majority of publicly available megamimivirus genome assemblies are fragmented or misassembled at their termini, capturing only a single TIR copy or failing to resolve the repeat entirely. Our recent work demonstrated that integrating third-generation long-read sequencing with short-read NGS data overcomes this obstacle, enabling the complete resolution of TIR structures (13). In *Megavirus baoshanense*, a member of the subfamily *Megamimivirinae*, the hybrid approach recovered 23-kb TIRs at both ends of the linear genome, whereas NGS-based assembly yielded only a single copy (13). This finding suggested that TIRs in many megamimiviruses have been overlooked, highlighting the need for a systematic reassessment of terminal structures across this subfamily.

In this study, we report the isolation and characterization of four new megamimivirus species isolated from diverse environments across China, including Megavirus daqingense (the first giant virus isolated from an oil reservoir), Megavirus honghaiwanense, Megavirus guangtanense, and Moumouvirus qiuqiense. Using a hybrid sequencing strategy, we resolved their complete linear genomes and identified long TIRs at both termini. We further conducted comparative genomics and synteny-based analyses on 42 published megamimivirus genomes, uncovering 29 distinct TIR structures within *Megamimivirinae*. Our analyses revealed that TIRs have high genetic diversity and are genomic hotspots for horizontal gene transfer. By elucidating the genetic complexity, selection pressures, and evolutionary dynamics of these terminal regions, our work provides a comprehensive view of TIRs as key drivers of genome plasticity and innovation in giant viruses.

## RESULTS

### Isolation, identification, and biological properties of four new megamimiviruses

**Fig 1:**
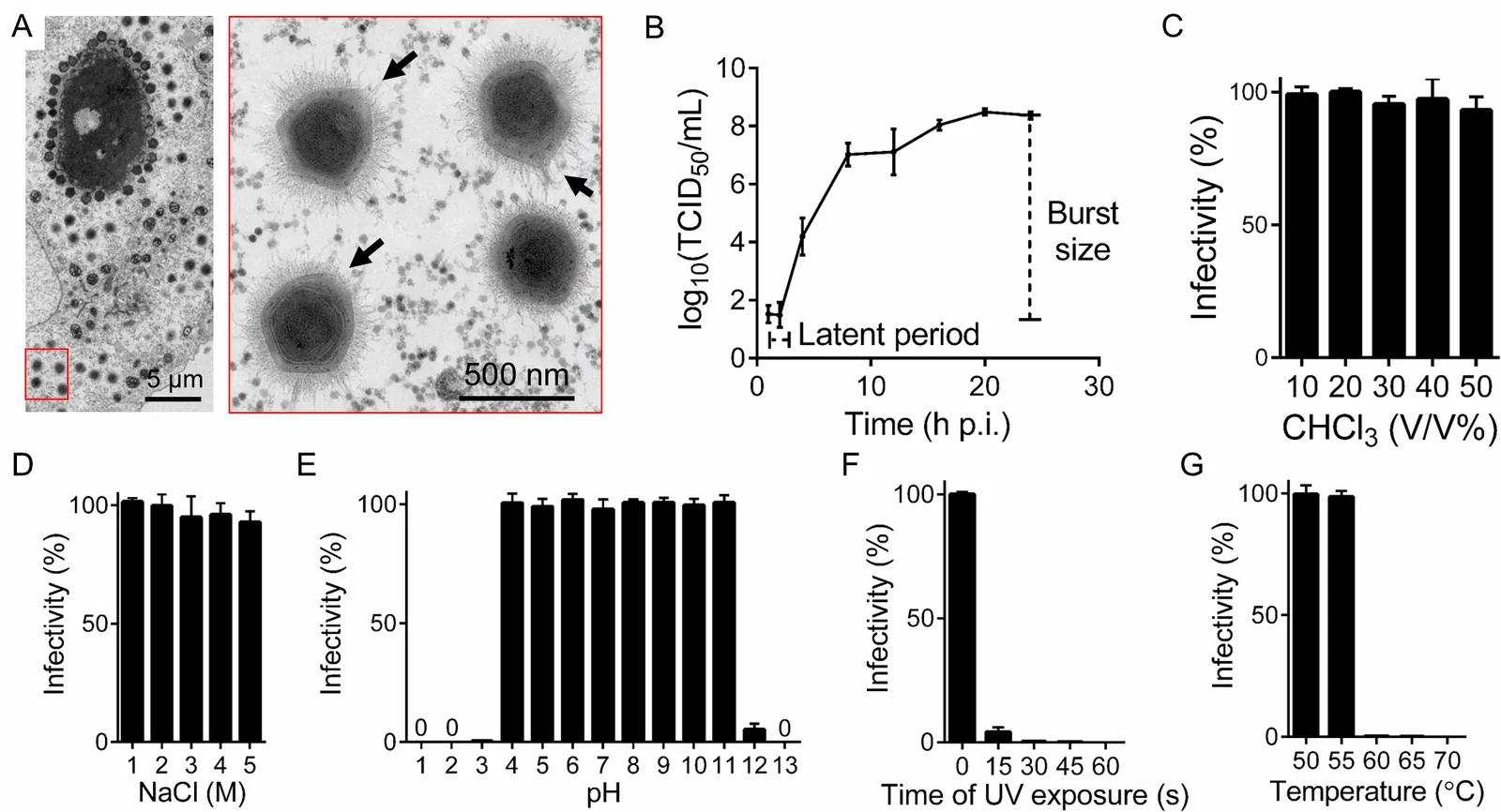
Biological properties of Megavirus daqingense. (**A**) Transmission electron microscopy images depicting the viral factory containing mature viral particles at 9 hours post-infection (h p.i.) and the stargate portal (black arrowhead) in the cytoplasm of infected *A. castellanii* cells. (**B**) Viral replication curve of Megavirus daqingense. TCID_50_, tissue culture infective dose 50. (**C–G**) Histograms displaying the percentage of infectious viruses after treatments with different concentrations of (**C**) chloroform, (**D**) NaCl, or (**E**) PBS at varying pH levels, (**F**) PBS under UV light after different exposure times, and (**G**) PBS at different temperatures. Data points represent mean values ± standard deviations from triplicate experiments.

From three geographically distant environmental sources (oil reservoir, mangrove wetland, and coastal bay) (Table S1), we successfully isolated and identified four new megamimiviruses based on transmission electron microscopy (TEM) and comparative genomics of whole-genome sequences. TEM analysis of infected *Acanthamoeba castellanii* cells revealed typical mimivirus-like virions with characteristic stargate faces and long surface fibrils within the cytoplasm (Fig. 1A; Fig. S1). The virion diameters of Megavirus daqingense, Megavirus honghaiwanense, Megavirus guangtanense, and Moumouvirus qiuqiense were 422 ± 5 nm (468 ± 7 nm including a stargate vertex), 417 ± 8 nm (468 ± 7 nm), 431 ± 3 nm (464 ± 12 nm), and 405 ± 16 nm, respectively. The corresponding surface fibril lengths were 95 ± 3 nm, 100 ± 2 nm, 93 ± 5 nm, and 79 ± 13 nm, respectively (Fig. 1A; Fig. S1).

Given its unique isolation from an oil reservoir, we conducted a detailed physiological characterization of Megavirus daqingense. The one-step growth curve revealed a replication cycle completed within approximately 14 h, yielding a burst size of 237 ± 24 infectious viral particles per infected amoeba (Fig. 1B). Physicochemical characterization revealed that Megavirus daqingense exhibited tolerance to chloroform treatment and high salinity (Fig. 1C and D) and a preference for alkaline conditions and sensitivity to acidic environments (Fig. 1E). Additionally, Megavirus daqingense was highly sensitive to UV irradiation, with a 15-s exposure resulting in a 95% decrease in infectivity (Fig. 1F). Similar biological properties were also seen in the other three isolates (Fig. S2A through D). Notably, Megavirus daqingense exhibited greater thermotolerance than its counterparts within the same genus (Megavirus honghaiwanense and Megavirus guangtanense), implying its adaptation to high-temperature subsurface environments (Fig. 1G; Fig. S2E).

### Genomic content and phylogenetic analysis

Using a hybrid assembly strategy combining short-read NGS (Illumina) and long-read sequencing (PacBio and MinION) platforms, we yielded complete genome maps for all four isolates, with genome sizes ranging from 1,067,849 to 1,265,278 bp (Table S2). GC contents were approximately 25.90%, consistent with previously reported megamimiviruses (13, 23, 24). The number of predicted protein-coding genes varied from 1,032 in Moumouvirus qiuqiense to 1,192 in Megavirus daqingense. Average intergenic distance varied from 98 nt (Moumouvirus qiuqiense) to 337 nt (Megavirus daqingense), resulting in coding densities ranging from 68.23% (Megavirus daqingense) to 90.58% (Moumouvirus qiuqiense). A total of 4,553 proteins, ranging from 29 to 2,912 amino acids with an average length of 221 residues, were predicted for the four new megamimiviruses. Comparative genomic analyses revealed substantial heterogeneity in their translation-associated gene repertoires, including aminoacyl-tRNA synthetase, tRNA, and translation factors.

**Fig 2:**
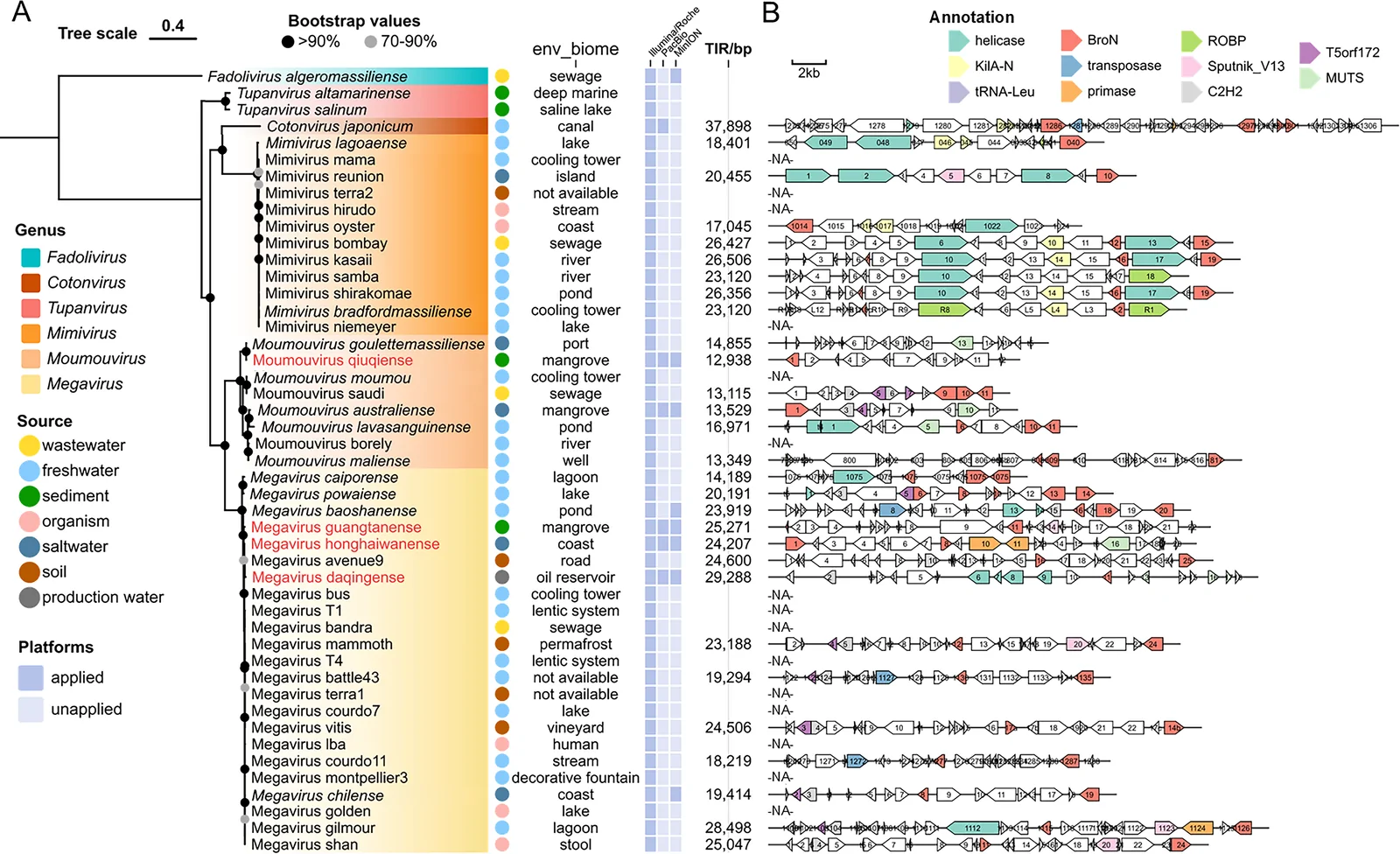
Molecular phylogenetic analysis of the subfamily *Megamimivirinae*. (**A**) Maximum likelihood phylogenetic tree based on concatenated amino acid sequences of five core genes of the phylum *Nucleocytoviricota* using *Fadolivirus algeromassiliense* (a member of the subfamily *Klosneuvirinae*) as an outgroup. Circles on each branch represent bootstrap values (percentages of trees supporting each branch). Background colors of megamimivirus names denote different genera. Solid symbols next to virus names indicate the environmental source of isolation, followed by the category of environmental origin (env_biome) and the genome sequencing platform. (**B**) Genomic synteny of gene loci across TIRs. Genes are labeled with locus tags and color-coded by gene function category. Scale bar, 2 kb. BroN, Bro-N domain-containing protein found in baculovirus; KilA-N, KilA-N domain-containing protein; V13, helicase V13 found in Sputnik virophage; ROBP, replication origin-binding protein; C2H2, C2H2-type zinc finger-N domain-containing; MUTS, MUTS family DNA-binding domain. NA (not available) indicates that no TIRs have been successfully identified.

**Fig 3:**
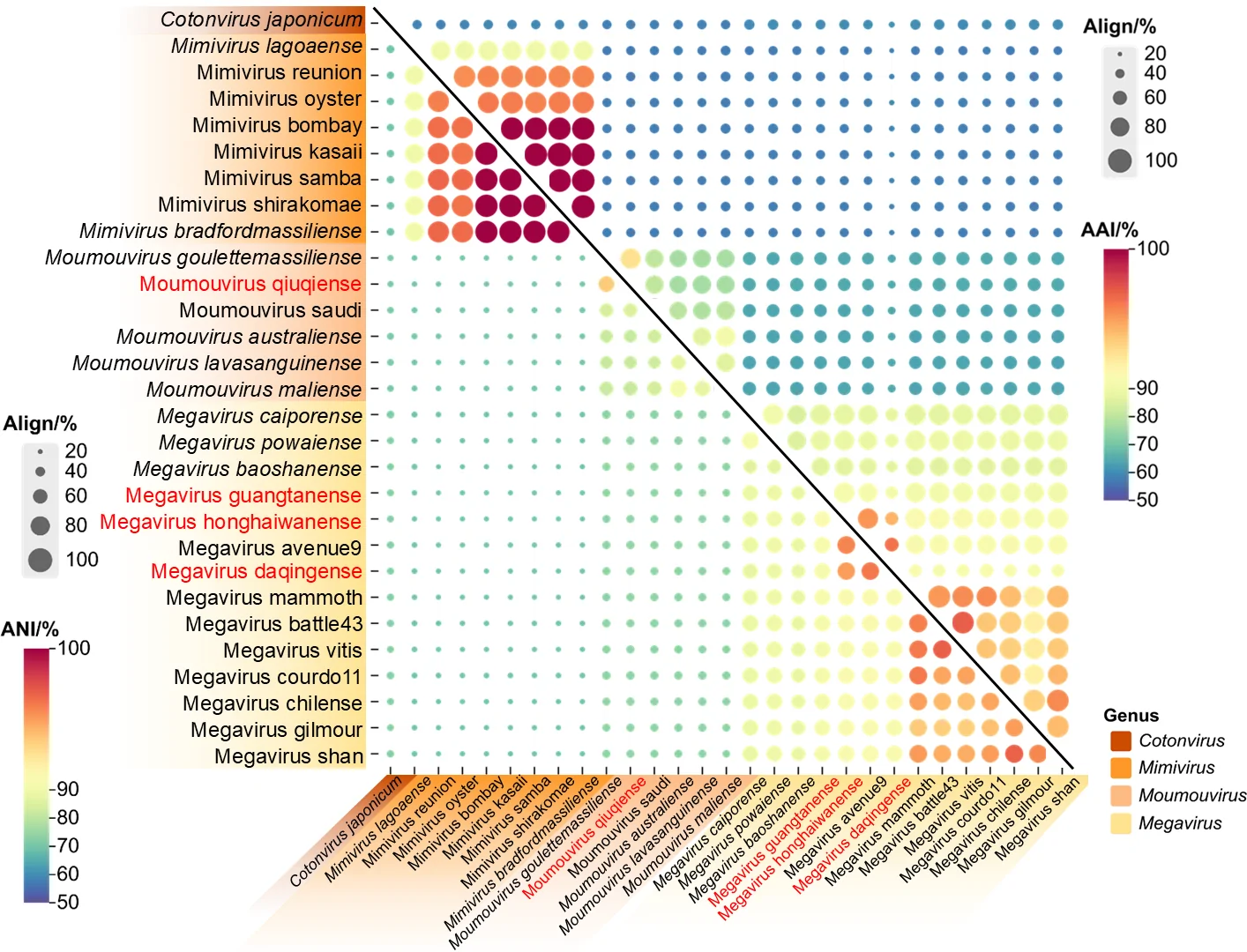
Bubble plot of pairwise ANI (lower-left) and AAI (upper-right) for megamimivirus genomes with the percentage of whole-genome alignment. ANI was calculated using JspeciesWS, and AAI was calculated using EzAAI. Different genera are indicated in different shades.

To determine the phylogenetic position of these four megamimiviruses within the family *Mimiviridae*, we performed phylogenetic analysis using five core genes of the phylum *Nucleocytoviricota* (see Materials and Methods). Currently, the subfamily *Megamimivirinae* includes five genera (*Mimivirus*, *Moumouvirus*, *Megavirus*, *Cotonvirus,* and *Tupanvirus*). The phylogenetic tree indicated that Megavirus daqingense, Megavirus honghaiwanense, and Megavirus guangtanense grouped within the genus *Megavirus*, whereas Moumouvirus qiuqiense clustered within the genus *Moumouvirus* (Fig. 2A). Average nucleotide identity (ANI) and average amino acid identity (AAI) analyses further confirmed their genomic divergence. Based on the widely used virus species demarcation threshold (pairwise ANI >95% across >75% of genes) (25), all four isolates were classified as new different species, a designation further supported by AAI values below 95% across more than 75% of shared genes (Fig. 3). Combining comparative genomics and phylogenetic analyses, we identified Megavirus daqingense, Megavirus honghaiwanense, Megavirus guangtanense, and Moumouvirus qiuqiense as four new species within the subfamily *Megamimivirinae*.

### Identification of TIRs within *Megamimivirinae*

**Fig 4:**
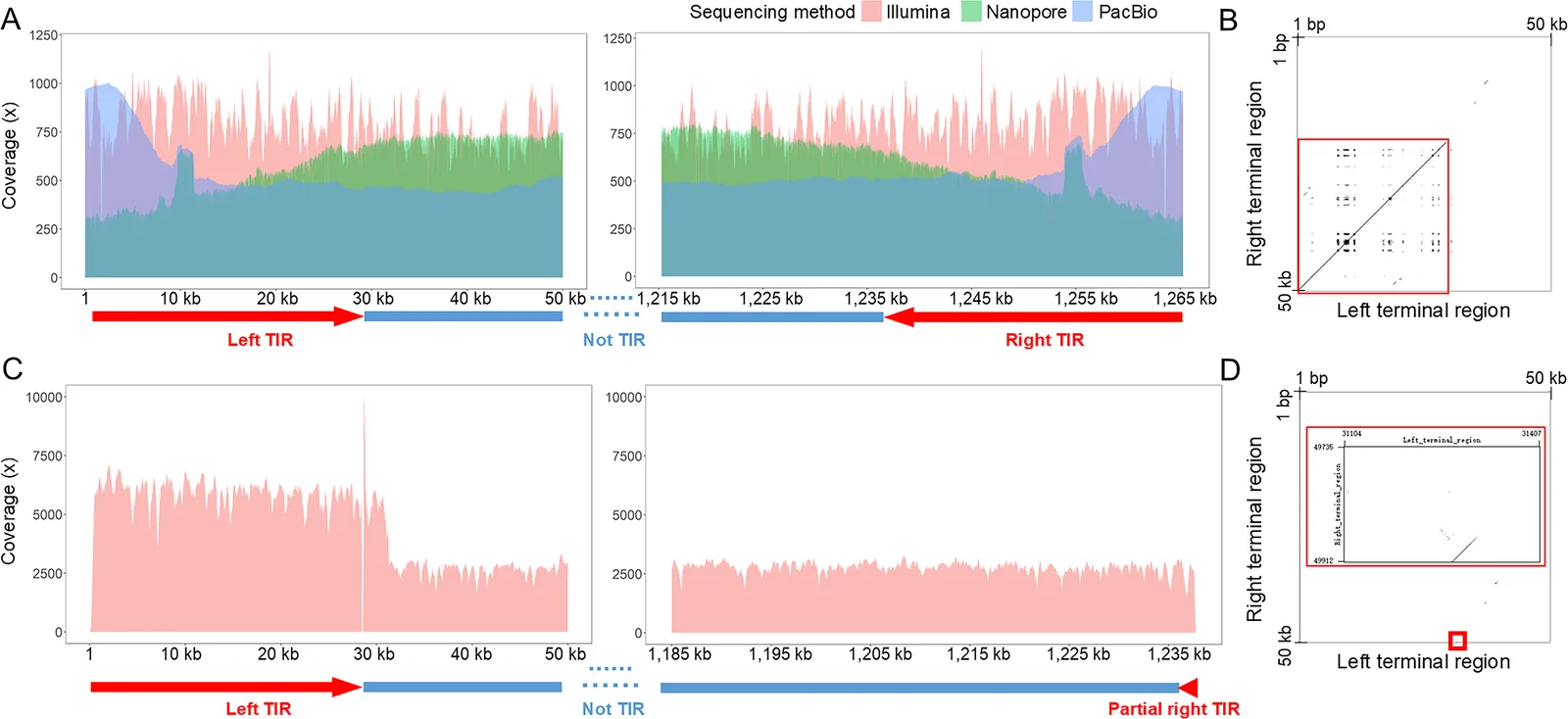
Coverage plots of the Megavirus daqingense genome by mapping clean reads from different sequencing platforms against (**A**) the complete genome map generated from hybrid sequencing and (**C**) the Illumina-based draft map assembled using SPAdes. Synteny analysis of the 50-kb left (*x*-axis) versus right (*y*-axis) terminal regions for (**B**) the complete genome map, where TIR is highlighted in the red frame, and (**D**) the Illumina-based draft genome map, where a short inverted repeat (i.e., a perfectly matched region) is highlighted in the red frame and enlarged in the inset.

Consistent with our previous findings in *Megavirus baoshanense* (13), hybrid sequencing technologies revealed the presence of TIRs at both extremities of the linear genomes of all four new isolates. Similar read distributions across different sequencing platforms and perfect synteny at terminal regions were observed (Fig. 4A and B; Fig. S3), indicating the ubiquity of TIRs among megamimiviruses.

Crucially, we identified a systematic misassembly artifact in Illumina-only assemblies. In the NGS-based drafts, the right TIR was frequently misassembled as a duplication of the left TIR, resulting in the erroneous identification of only a single TIR copy. Additionally, an approximately 150-bp perfectly matched palindromic sequence was detected near the right end region of the Megavirus daqingense genome (Fig. 4C and D). Given that most public megamimivirus genome assemblies are NGS-based and generated with different assembly software, we investigated whether this misassembly was consistent across assemblers by reconstructing the Megavirus daqingense genome using multiple assembly tools. Read remapping confirmed that the misassembly pattern and short palindromic sequences were consistently reproduced across all tested NGS-based assemblers (Fig. S4), demonstrating that TIRs are commonly misrepresented in short-read assemblies. We further analyzed the inverted repeats in the NGS-based draft maps of *Mimivirus bradfordmassiliense* (2), revealing a 600-bp perfect inverted repeat near the right end, while the corresponding left TIR was located 22 kb away from the left terminus. The length of these short inverted repeats was close to the insert size of NGS libraries (26), and thus, they were retained. Therefore, the short perfectly matched inverted repeats can be used to indicate the positions of TIRs. We then extended the analysis to 42 previously published megamimivirus genomes by searching for perfectly matched inverted repeats. This allowed us to successfully identify 25 TIRs, ranging from 12 to 37 kb in length, across 25 of these genomes (Fig. 2; Fig. S5 to S7).

### TIR characterization and diversity

The 29 identified TIRs, comprising 25 from public genomes and 4 from isolates in this study, exhibited distinct gene content. Their average GC content was 22.93% ± 0.74%, which is significantly lower than that of the corresponding whole genomes (25.90% ± 0.23%). These TIRs displayed significantly lower inter-sequence similarity than both whole genomes and non-TIR regions across different species within the genera *Mimivirus* and *Megavirus* (Fig. S8A). Sequence alignments revealed a lack of conservation between TIR and non-TIR regions across different species within *Megamimivirinae* or within the same genus (Fig. S8B). Meanwhile, unlike non-TIR regions containing highly conserved regions (Fig. S9), no conserved regions were identified across the TIRs of the subfamily *Megamimivirinae*; genus-specific analysis showed scattered conserved regions, with four regions in *Mimivirus*, one in *Megavirus*, and none in *Moumouvirus* TIRs (Fig. S10).

**Fig 5:**
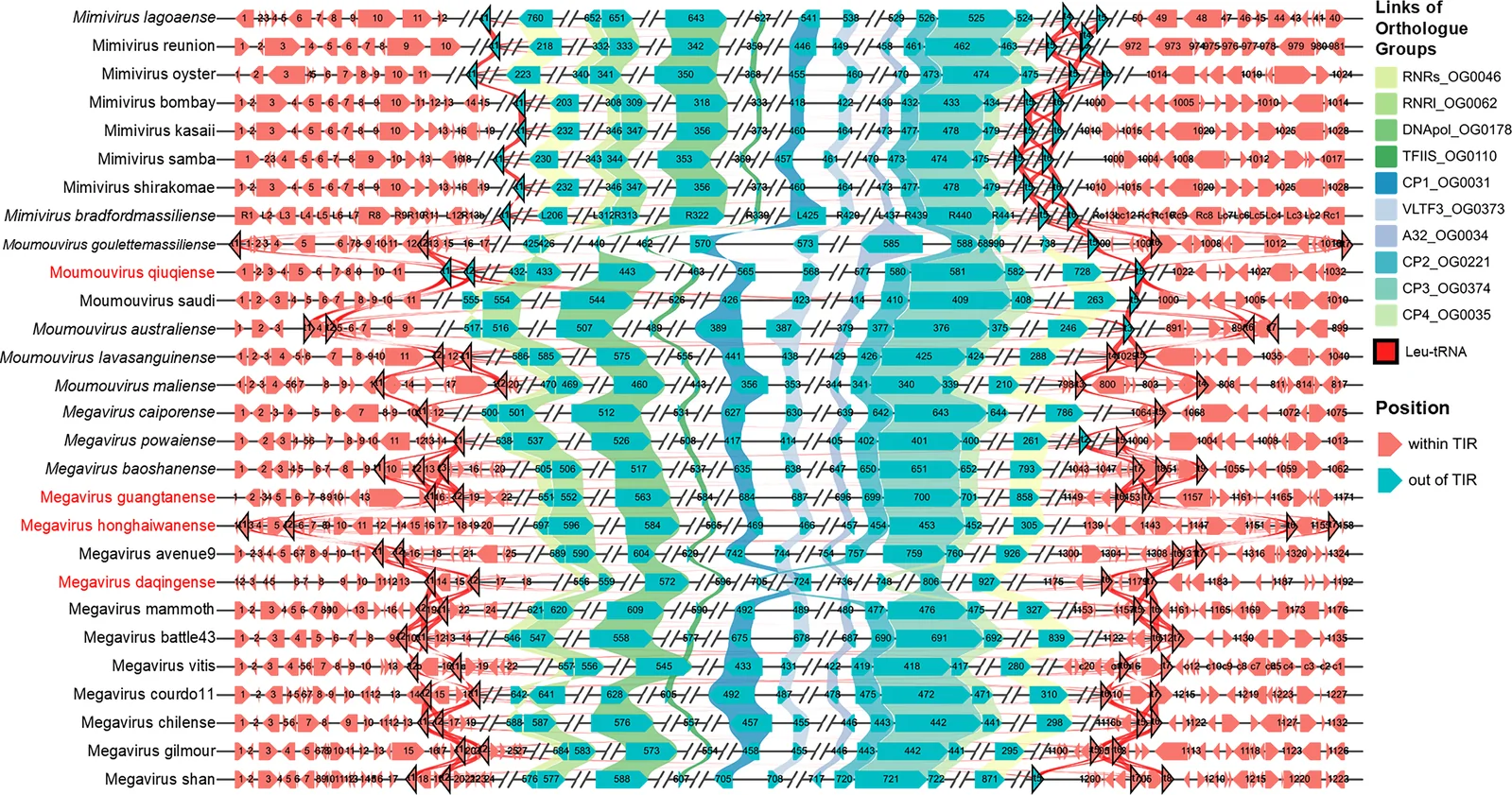
Map of megamimivirus TIRs and selected non-TIRs, displaying the direction and position of ORFs, and Leu-tRNA genes highlighted in bold arrows. Colored links indicate homologous proteins shared among different viruses. RNRs, small subunit of ribonucleotide reductase; RNRl, large subunit of ribonucleotide reductase; DNApol, family B of DNA polymerase; TFIIS, transcription factor S-II; CP1–CP4, capsid proteins 1 to 4; A32, A32-like packaging ATPase; VLTF3, poxvirus late transcription factor VLTF3.

In each TIR, 11 to 25 open reading frames (ORFs) were predicted, of which approximately 33% (39/110) were assigned known functional annotations. Unlike whole-genome analysis, which identified 260 core orthologous protein clusters shared by all 29 megamimiviruses, no core orthologous protein cluster was shared among TIR genes across the 29 viruses, despite the presence of some orthologous proteins (e.g., Bro-N domain-containing proteins) across 13 of the viruses (Fig. S11). Additionally, one to three copies of tRNA genes (i.e., Leu-tRNA genes) were predicted in the *Moumouvirus* and *Megavirus* TIRs. Genomic synteny analyses of TIR gene loci indicated a diverse protein repertoire and extensive gene rearrangements across virus species (Fig. 2B; Fig. S5). Among the 39 functionally annotated orthologous protein clusters, the largest included Bro-N domain-containing proteins, followed by C2H2-type zinc finger proteins, DEAD-like helicases, MUTS family proteins, transposase, and KilA-N domain-containing proteins. Most of these are DNA-binding proteins potentially involved in DNA recombination, replication, and repair. Further gene-position analysis revealed that orthologs of TIR genes from one species could be found in the non-TIR regions of other species (Fig. 5; Fig. S12A). For instance, the transposases encoded by the TIR of *Megavirus baoshanense* were orthologs to those in the non-TIR region of Mimivirus reunion (Fig. S12B). These results indicated that gene recombination events may have occurred between TIRs and/or non-TIR regions across different species.

**Fig 6:**
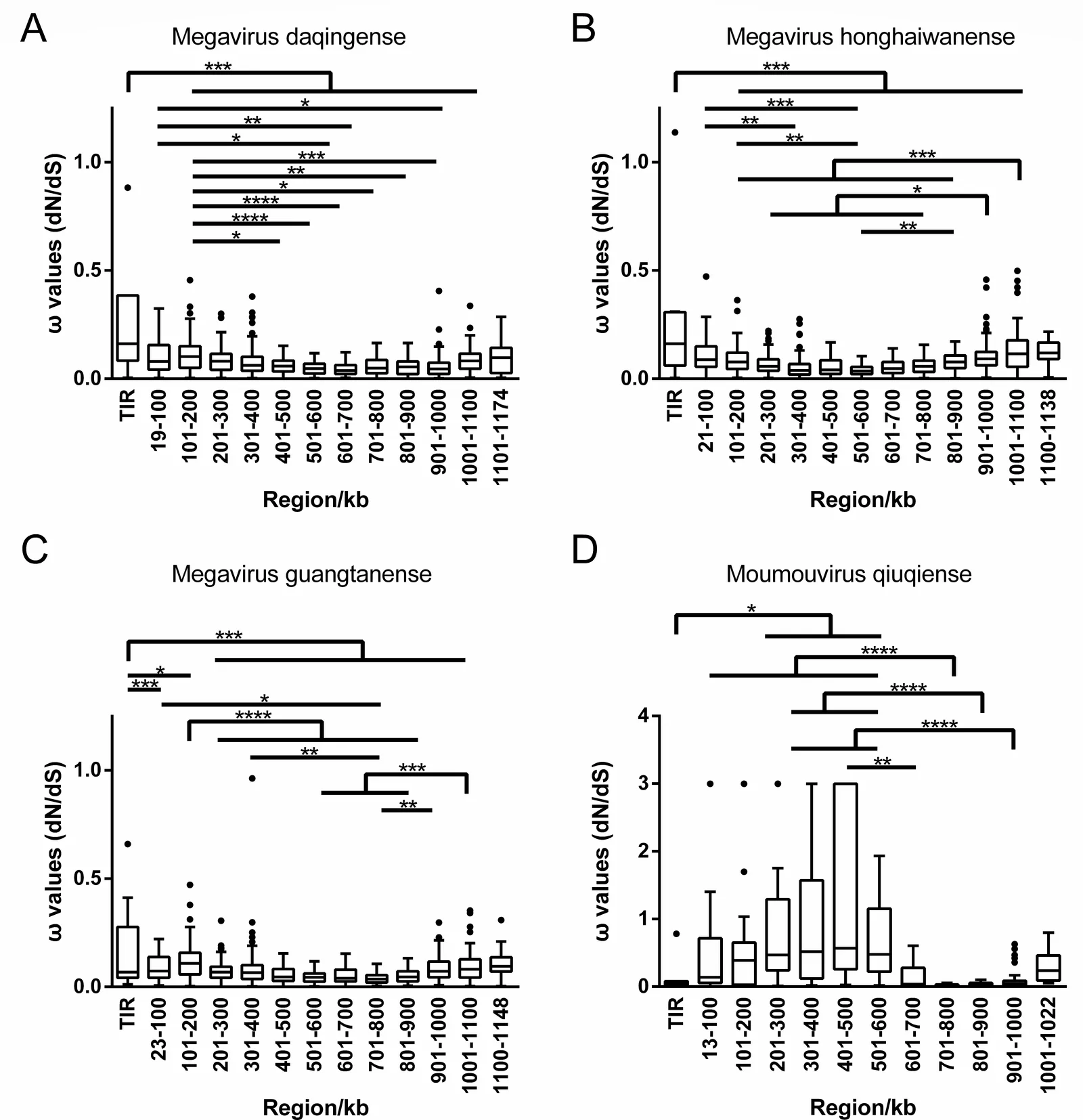
Distribution of ω-values (dN/dS) along linear genomes of (**A**) Megavirus daqingense, (**B**) Megavirus honghaiwanense, (**C**) Megavirus guangtanense versus *Megavirus baoshanense*, and (**D**) Moumouvirus qiuqiense versus *Moumouvirus australiense*. The *x*-axis represents the gene positions along the linear genome. Asterisks denote statistical significance using the two-tailed Mann–Whitney test: *, **, ***, and **** indicate *P* value lower than 0.05, 0.01, 0.001, and 0.0001, respectively.

We next assessed gene selection pressure by comparing our isolates with previously characterized relatives and by comparing different genomic regions (TIRs versus non-TIR regions). More than 80% ORFs (3,648/4,553) of the four isolates were found to be under purifying selection, as indicated by the average ω values (dN/dS, the ratio of nonsynonymous rates [dN] to synonymous rates [dS] substitutions). Notably, significantly higher ω and dN were observed for genes in TIRs than in non-TIR regions in the genus *Megavirus*, indicating weaker purifying selection on genes in TIRs. Similarly, significant differences in ω and dN were observed when comparing genes adjacent to TIRs with those located near the central region (Fig. 6; Fig. S13). These differences in region-dependent selection pressure indicated that genes within TIRs and in regions proximal to TIRs experience significantly weaker purifying selection than genes in central regions, despite relatively uniform substitution rates across whole genomes.

### Distribution and evolutionary dynamics of tRNA genes in megamimiviruses

**Fig 7:**
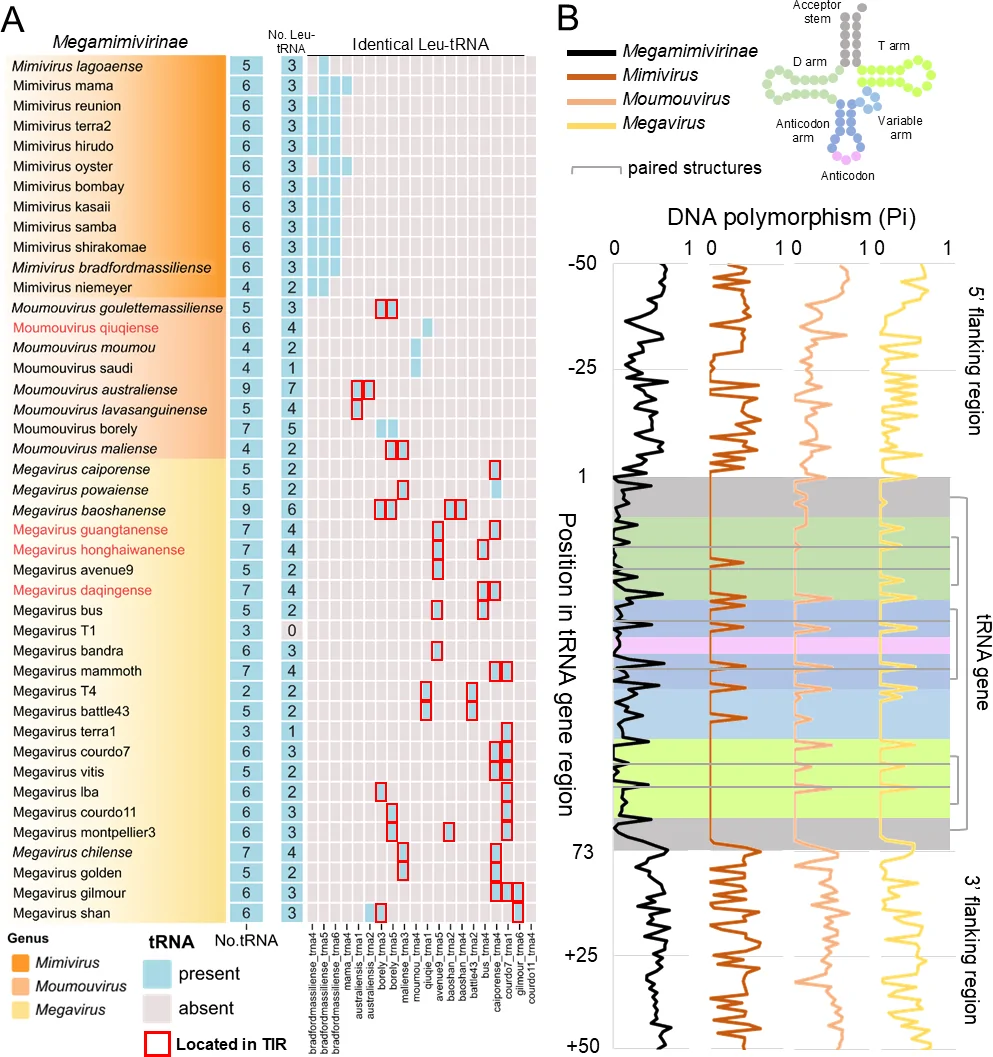
Conserved Leu-tRNA in megamimiviruses. (**A**) Presence of identical tRNAs across different megamimiviruses. Numbers in aquamarine cells represent the copy number of tRNAs in the published genome of each virus, followed by that of Leu-tRNAs. The plot represents the presence or absence of Leu-tRNA genes, with red rectangles indicating those located within TIRs. The *x*-axis labels correspond to representative Leu-tRNA gene names, for which detailed information is provided in Fig. S16A. (**B**) Variation across Leu-tRNA gene regions in megamimiviruses. The *x*-axis shows DNA polymorphism levels calculated by DnaSP (1-bp moving window average), and the *y*-axis indicates position within the tRNA gene regions. Regions within the tRNA gene are color-coded according to secondary structure, as shown in the schematic diagram at the top right, with brackets connecting paired structural elements. The schematic diagram is colored and labeled by the component of the tRNA “cloverleaf” structure.

Genome-wide analysis revealed that megamimiviruses encode a variable repertoire of tRNAs, with copy numbers ranging from three to nine per genome (Fig. 7A). Among the different tRNA types, Leu-tRNA was commonly present in the highest copy number (Fig. 7A; Fig. S14A). Notably, while other tRNA genes were exclusively located in non-TIR regions, Leu-tRNA genes exhibited different genomic locations across genera. Specifically, nearly all *Megavirus* Leu-tRNA genes (54/56) and the majority of *Moumouvirus* Leu-tRNA genes (18/23) were located in TIRs, whereas all *Mimivirus* Leu-tRNA genes (35/35) were found in non-TIR regions.

Interestingly, identical Leu-tRNA sequences were observed across the TIRs of different viral species within the genus *Megavirus* (Fig. S15A), while in some cases, identical Leu-tRNA genes were also shared between TIRs and non-TIR regions across genera (Fig. 7A). For instance, a Leu-tRNA gene present in the TIRs of Megavirus battle43 and Megavirus T4 was found identically in the non-TIR region of Moumouvirus qiuqiense, with variant 5′ and 3′ flanking regions (Fig. S15B), implying a virus-to-virus horizontal gene transfer event (vHGT) (27) between TIR and non-TIR regions across these genera. By contrast, while identical sequences of other tRNAs (i.e., Cys-tRNA, His-tRNA, and Trp-tRNA) are also shared among multiple viral species, these three tRNA types are always located in non-TIR regions rather than in TIRs (Fig. S14A).

DNA polymorphism analysis revealed that megamimivirus tRNA genes exhibit significantly lower polymorphism compared to their flanking regions. This pattern was consistent across all megamimiviruses and within individual genera (*Mimivirus*, *Moumouvirus*, and *Megavirus*). The conservation was particularly pronounced in sequences essential for forming the stereotypical cloverleaf secondary structure (Fig. 7B; Fig. S14B through D), indicating stronger purifying selection.

**Fig 8:**
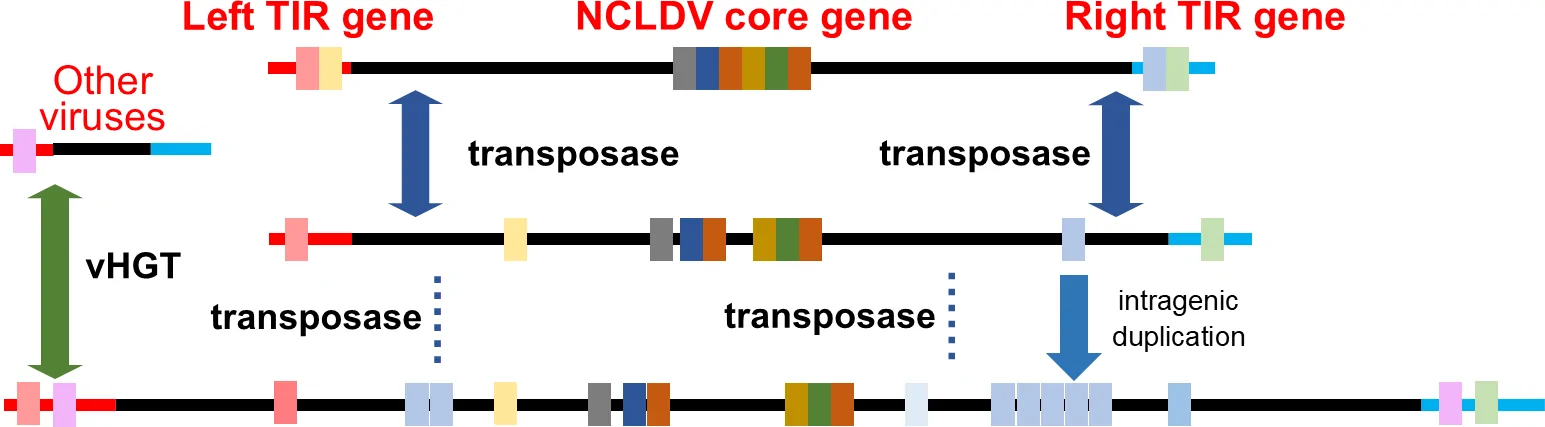
Schematic model of the proposed accordion-like expansion mechanism for the megamimivirus linear genomes. In the megamimivirus linear genome, NCLDV core genes were vertically inherited from the ancestral virus. Meanwhile, TIR genes, whether vertically inherited or vHGT from other viruses, were mobilized between TIR and non-TIR regions via transposase activity. These reversible events facilitated the translocation of dispensable genes from non-TIR regions into TIRs, where they were subsequently lost. Transposase activity continually drives these genomic dynamics, leading to gene gain/loss, intragenic duplication, and chromosomal rearrangements. Consequently, the megamimivirus linear genome evolves dynamically through cycles of expansion and reduction, with TIRs serving as critical hotspots for these rearrangements.

To infer evolutionary trajectories of megamimivirus tRNA genes, phylogenetic analyses were performed for different tRNA types. Leu-tRNAs encoded by *Megavirus* and *Moumouvirus* shared a close evolutionary relationship, with sequences from those genera often clustering together (Fig. S16A). In contrast, the phylogenetic relationships of the other three tRNAs mirrored the phylogeny based on concatenated *Nucleocytoviricota* core genes, forming monophyletic clades that corresponded to the genus classification (Fig. S16B through D). The different evolutionary trajectories between Leu-tRNAs and other tRNAs (located in non-TIRs) suggest the preference for vHGT events occurring in TIR regions. This observation is further supported by the similar phylogenetic topology reported previously (28), which indicates that the major clade relationships identified here are reproducible for these genes, albeit with moderate bootstrap support at a few nodes, a limitation likely attributable to the inherently short sequence length and structural conservation of tRNA (29). Furthermore, while individual megamimiviruses often encoded multiple Leu-tRNA genes, these copies are not identical. For example, in each of our three *Megavirus* isolates, the Leu-tRNA encoded within a single TIR differed by 2 to 10 single-nucleotide polymorphisms (SNPs) (Fig. S17A through C). Nevertheless, identical orthologs of these genes can be found across different megamimiviruses, supporting the role of TIRs as hotspots for vHGT events (Fig. 5 and 8). In Moumouvirus qiuqiense, four Leu-tRNA genes, which are all located in the non-TIR region, differ from one another by two to eight SNPs. Only one of them has an identical gene in the TIR of Megavirus battle43 (30), indicating a recent vHGT event. The other three Leu-tRNA copies are divergent paralogs that likely arose from gene duplication events following an earlier vHGT (Fig. S17D).

## DISCUSSION

Co-culture techniques combined with metagenomic surveys have previously shown that megamimiviruses are globally distributed, inhabiting a wide range of environments, including permafrost, artificial habitats, mangroves, rivers, and oceans (31, 32). We add to this picture by isolating four new giant virus species from distinct habitats in China (oil reservoir, mangrove wetland, and coastal bay). Notably, Megavirus daqingense is the first giant virus isolated from an oil reservoir, which is an extreme environment characterized by high pressure, high salinity, high temperature, low redox potential, and limited nutrients (33, 34). Our isolation work significantly expands the known geological distribution range of giant viruses and suggests that similar extreme niches may harbor undiscovered viral diversity.

Comparative genomic analyses reveal that megamimiviruses share architectural similarities with other large DNA viruses in *Nucleocytoviricota,* such as poxviruses and African swine fever virus (ASFV), with conserved central regions housing essential core genes and being flanked by diverse terminal regions that encompass TIRs (18, 19). However, in poxviruses, the TIR genes are often involved in immune evasion and host range determination (35), whereas TIR genes of megamimiviruses predominantly encode DNA recombination, replication, and repair proteins (e.g., Bro-N domain, helicases, and transposases). This functional divergence suggests that TIRs have evolved distinct roles across different viral families, likely reflecting lineage-specific adaptations of TIR functions.

Our data further reveal that megamimivirus TIRs serve as dynamic interfaces for genetic exchange. Specifically, we identified potential vHGT events between TIRs and between TIRs and non-TIR regions across species, analogous to gene acquisition in bacterial linear chromosomes and plasmids (e.g., *Streptomyces* species) (36, 37). Although information on the TIRs of Mimivirus strain M4 remains incomplete, the previously reported gene loss, low gene expression, and gene inactivation that occurred at both ends of the genome likely reflect the fate of TIR genes (9, 38). In addition, in four of seven complete megamimivirus genome maps (our four isolates and three previously published maps [13, 20, 39]), we noticed that a gene at the TIR–non-TIR boundary was present only at one terminus (data not shown), hinting that novel genes may emerge from TIR regions. These observations support the “genomic accordion” model of viral genome evolution (10), wherein TIRs may act as hotspots for gene gain, loss, and emergence (Fig. 8). Selection pressure analyses provided additional support for the role of TIRs as evolutionary hotspots, revealing significantly weaker purifying selection on genes within and proximal to TIRs than on non-TIR genes. Similar region-dependent selection pressures have been found in *Mollivirus* (40), *Orthopoxvirus* (41), and *Streptomyces* species (42), suggesting that reduced selective constraint on terminal regions may be a general feature of linear genomes. Such relaxation likely facilitates the rapid evolution of TIR-associated genes, thereby enabling adaptation to changing environments or hosts. New genes, such as Leu-tRNA or those that function to modify the host response (41), were potentially transferred from different resources and mobilized into the non-TIR regions by transposase, followed by gene duplication, driving genome expansion (Fig. 8).

In eukaryotes, tRNA genes in the same isoacceptor family, which accept the same amino acid, are encoded by multiple nuclear genes, many of which are completely identical in sequence. These paralogous tRNA genes are distributed in different genomic locations (43, 44). However, unlike in eukaryotes, paralogous Leu-tRNA genes within the single megamimivirus genome showed variant SNPs, reflecting the rapid evolutionary rate of viruses (45). On the other hand, identical tRNA genes are found across the genomes of different species in eukaryotes (44, 46), prokaryotes (47), and bacteriophages (48). Herein, we observed identical Cys-tRNA, His-tRNA, and Trp-tRNA genes across different megamimivirus species, suggesting a common ancestor. Meanwhile, identical Leu-tRNA genes showed both vertical inheritance and multiple independent vHGT events that happened between *Megavirus* and *Moumouvirus* or within *Megavirus*. This pattern suggests that Leu-tRNAs within TIRs are under relatively strong selective constraints, despite their location in highly variable genomic regions and vHGT hotspots. Similarly, in diverse tRNA clusters encoded by 41 chloroviruses (49), we also found identical Ile-tRNAs across different clades (data not shown), indicating the ubiquity of identical tRNA genes via vertical inheritance or vHGT in giant viruses. Furthermore, phylogenetic analyses of megamimiviruses and host amoeba tRNA genes (data not shown) confirmed the distant evolutionary relationship between amoeba and giant viruses (28) and support the hypothesis that mimivirus tRNAs do not seem to originate from currently known amoeba hosts (50).

In summary, by applying a hybrid sequencing approach, we resolved complete TIR structures for four new isolates. Through a bioinformatic screen for perfectly matched inverted repeats, we further identified 25 additional TIRs across 42 previously published genomes. Investigation of terminal structures of megamimivirus linear genomes revealed TIRs as hotspots of genetic diversity. Genes within TIRs exhibited significantly weaker purifying selection pressure than non-TIR genes, consistent with their role as drivers of genome plasticity. Within these diverse TIRs, we report the first identification of conserved Leu-tRNA genes across different species and genera. By integrating insights into giant virus gene diversity and conservation, our work advances understanding of giant virus evolution and highlights TIRs as crucial architects of linear viral genomes. Furthermore, we demonstrate that short-read NGS assemblies systematically misrepresent TIRs of giant viruses. Nearly half of the examined published genomes still lack resolvable TIRs, likely due to assembly fragmentation. Future studies presenting high-quality linear genomes of giant viruses will fully characterize the TIRs and TIR–non-TIR boundary and substantially expand our understanding of large viral genomes.

## MATERIALS AND METHODS

### Giant virus isolation and purification

*A. castellanii* strain Neff (ATCC 30010) was cultivated in peptone-yeast extract with glucose medium (PYG) at 27°C for 2 days, and then, the medium was replaced with Page’s amoeba saline (PAS) containing 50 mg/L thiabendazole, 10 mg/L vancomycin, 20 mg/L ciprofloxacin, and 20 mg/L rifampicin. Water samples from the Daqing oilfield, Zhangjiang mangrove reservoir, and Red Bay were collected in Heilongjiang, Fujian, and Guangdong provinces, China, respectively. The detailed locations are listed in Table S1. The protocols of sample processing, megamimivirus isolation, cloning, titration, and burst size have been described previously (13, 40).

### Chloroform sensitivity, pH, saltiness, temperature, and UV exposure stability assays

Environmental tests, including stability under different chloroform concentrations, pH levels, temperatures, and salinity levels, were conducted as described in our previous work (51). Briefly, the purified virions were resuspended in phosphate-buffered saline (PBS). For chloroform sensitivity, the virus solutions were mixed with chloroform at room temperature. The mixture was centrifuged, and the aqueous phase was transferred to a new sterile tube and then incubated for 12 h to remove any remaining chloroform. For pH stability measurements, the virus solutions were adjusted to the desired pH and incubated for 12 h. For salt stability, the purified virus pellets were resuspended in PBS containing the desired concentration of NaCl and incubated for 12 h. For thermal stability tests, the virus solutions were incubated in a GeneAmp 2400 thermal cycler (Perkin-Elmer-Cetus, CT, USA) at different temperatures for 1 h. After the different treatments, virus solutions were centrifuged to collect pellets, resuspended in PBS, and assayed. For UV exposure stability assays, the virus solutions were centrally spotted onto a sterile Petri dish and immediately sealed with parafilm to prevent aerosol contamination. The dish was positioned directly at 10 cm beneath a 30 W UV-C lamp (wavelength: 254 nm) and exposed for predetermined durations. The treated samples were promptly transferred into sterile tubes and wrapped entirely in aluminum foil to block any residual light exposure.

### Transmission electron microscopy

The amoeba cells infected with viruses at a multiplicity of infection (MOI) of 1 were harvested by centrifugation at 356 × *g* for 5 min and washed twice with PBS. The amoeba cells, which were fixed with 2.5% glutaraldehyde and stained with 2% osmium tetroxide, were then processed with embedding and ultrathin sectioning using an ultramicrotome (Leica, Wetzlar, Germany). The meshes were stained with 2% uranium acetate, avoiding light for 8 min, and 2.6% lead citrate. After the meshes were dried at room temperature for 12 h, observations were performed using a Hitachi HT7800 transmission electron microscope (Tokyo, Japan). The TEM results were analyzed using ImageJ v 1.54f with the measure function (52).

### DNA preparation and genome sequencing

Genomic viral DNA was prepared from the supernatant containing cloned megamimivirus using PureLink Genomic DNA Mini Kit (Invitrogen, USA) following the manufacturer’s protocol. Viral DNA was quantified by fluorometric quantitation with a Qubit 3.0 Fluorometer (Thermo Fisher Scientific, USA). High-quality genomic DNA (2 μg) was used for DNA Illumina sequencing, which was carried out by GENEWIZ Technologies Inc. (Suzhou, China). Viral DNA was fragmented into 400–500 bp using the Covaris M220 ultrasonic instrument. The DNA library was prepared using TruSeq DNA Sample Prep Kit (Illumina, CA, USA) and sequenced on an Illumina NovaSeq PE150. Nanopore sequencing was performed following the manufacturer’s protocol (Nanopore, Oxford, UK). For PacBio sequencing, the DNA library was prepared using an SMRTbell Express Template Preparation Kit 2.0 (Pacific Biosciences, CA, USA), and sequencing was performed on a PacBio RS II sequencer. Briefly, the sheared fragments were treated with single-strand overhangs, DNA damage repair, end repair, A-tailing, and ligation of barcoded overhang adapters. The library was quantified using Qubit, and the size of the library was checked using an Agilent 2100 Bioanalyzer System (Agilent Technologies, CA, USA). The library was subsequently sequenced on the PacBio Sequel platform.

### Genome assembly and annotation

Illumina and Nanopore paired-end raw reads were processed to obtain clean reads, as described in previous works (13). Briefly, after quality control of reads, the Illumina paired-end clean reads were assembled *de novo* using SPAdes v3.15.2 (53), SOAPdenovo2 v2.04 (54), Velvet v1.2.10 (55), A5-miseq v20160825 (56), and ABYSS v2.3.10 (57) with K-mer 95 to obtain the contigs. Contigs were aligned with the complete map using ragoo v2.30-r1287 (58) to obtain the draft assemblies. The Nanopore clean reads were processed and recorrected by Illumina clean reads to obtain the complete maps (13). The clean reads of Illumina, Nanopore, or PacBio were mapped on the draft assemblies or complete maps. The visual exploration of reads-mapping data was conducted with the R package ggplot2 v3.5.1 (59). Protein-coding sequences (CDSs) were identified using the GeneMarkS v4.28 with virus sequence type (60). Transfer RNAs were found using tRNAscan-SE v2.0.9 (61). The functional assignment of predicted genes was performed using the BLASTP search against the non-redundant (NR) protein sequences database with an *E*-value cutoff of 1e−05.

### Phylogenetic analysis

The megamimivirus genomes in the subfamily *Megamimivirinae* and *Fadolivirus algeromassiliense* were retrieved from GenBank, and their accession numbers are listed in Table S3. Orthologue sequences were extracted from the results of ProteinOrtho v6.3.1 (BLASTP: *E*-value = 1e−05, coverage = 50, and identity = 25), and the results were visualized using proteinortho_curves (62). Five core genes among nucleocytoplasmic large DNA viruses (NCLDV) (i.e., major capsid protein 1, family B DNA polymerase, D5-like primase-helicase, transcription factor S-II, and virion packaging ATPase) (3) were chosen for building the phylogenetic trees. Orthologue amino acid residue sequences were aligned using MAFFT v7 (63). Phylogenetic trees of NCLDV core proteins based on amino acid sequences were generated using IQ-TREE multicore v2.1.2 (64), and the best evolutionary models were calculated to be the LG+F+R4 model according to the Bayesian information criterion (BIC) value and 1,000 bootstrap replicates for the concatenated NCLDV core proteins. Phylogenetic trees of tRNAs based on nucleotide sequences found by tRNAscan-SE were generated using IQ-TREE, and the best evolutionary models were calculated to be HKY+G4, HKY+I, TPM2u+I+G4, and K3P+G4 models for Cys-tRNA, His-tRNA, Leu-tRNA, and Trp-tRNA, respectively, according to BIC value and 1,000 bootstrap replicates for each tRNA gene. Phylogenetic trees were visualized using Chiplot v2.6 (65).

### Comparative genomics analysis

The ANI was calculated using JSpeciesWS v4.2.1 based on BLAST (66), and the AAI was calculated using EzAAI v1.2.2 (67). Both ANI and AAI results were visualized using Chiplot. Two long regions of the left and right terminals of the complete or draft assemblies were compared using BLAST (68). Synteny analyses of different Mimivirus, Megavirus, and Moumouvirus genomes were performed using Gepard v1.40 (69) and Mauve v20150226 (70). The ratio (dN/dS) was computed by PAML_X v1.3.1 using the YN00 program with default options (71). The tRNA genes were aligned using MAFFT and visualized using the MultAlin website (72). The DNA polymorphism indices were calculated using DnaSP v6.12.03 (73), and secondary structure information for megamimivirus tRNA was extracted from the results of ARAGORN v1.2.41 (74).

### Statistical analysis

Multiple-group comparisons were performed and visualized using GraphPad Prism v6.01 (https://www.graphpad.com). The statistical significance for stability value (Fig. S1) and sequence similarity/alignment percentage (Fig. S8) were assessed using the unpaired *t*-test.

## Data Availability

The annotated genomes of Moumouvirus qiuqiense, Megavirus honghaiwanense, Megavirus guangtanense, and Megavirus daqingense were deposited in the GenBank database under accession numbers PX691301.1, PQ010613.1, PX691300.1, and MT663335.2, respectively. The raw DNA sequencing data were deposited into the CNGB Sequence Archive (CNSA) of China National GeneBank DataBase (CNGBdb) (75) with accession numbers CNS1311082, CNS1219738, CNS1311086, and CNS1224501, respectively.

## SUPPLEMENTAL MATERIAL

Supplemental material jvi.00825-26-s0001.docx Fig. S1 to S17; Tables S1 to S3.

ASM does not own the copyrights to Supplemental Material that may be linked to, or accessed through, an article. The authors have granted ASM a non-exclusive, world-wide license to publish the Supplemental Material files. Please contact the corresponding author directly for reuse.
