# Supplementary material for: Megamimivirus double-stranded DNA linear genomes flanked by highly diverse terminal inverted repeats
Source: J Virol. 2026 Sep 9;100(10):e00825-26. doi: 10.1128/jvi.00825-26 (PMC13712841; doi:10.1128/jvi.00825-26)
Supplement: Supplementary file 1 [file jvi.00825-26-s0001.docx]

**Supplementary Information for**

**Title:**	Megamimivirus double-stranded DNA linear genomes flanked by highly diverse terminal inverted repeats

**Running title:** Terminal inverted repeats in megamimivirus genomes

**Authors:**	Yucheng Xia^1^, Lanlan Cai^1*^, Boyu Ding^2^, Baiyu Su^2^, Rui Zhang^2*^

**Affiliation:** ^1^ Earth, Ocean and Atmospheric Sciences Thrust, The Hong Kong University of Science and Technology (Guangzhou), Guangzhou, 511453, China.

^2^Archaeal Biology Center, Synthetic Biology Research Center, Shenzhen Key Laboratory of Marine Microbiome Engineering, Key Laboratory of Marine Microbiome Engineering of Guangdong Higher Education Institutes, Institute for Advanced Study, Shenzhen University, Shenzhen 518055, China.

***** Correspondence to Lanlan Cai ([lanlancai@hkust-gz.edu.cn](mailto:lanlancai@hkust-gz.edu.cn)) or Rui Zhang ([ruizhang@szu.edu.cn](mailto:ruizhang@szu.edu.cn))

# Figures and Tables

**Fig. S1.** Transmission electron microscopy images of (A) Megavirus honghaiwanense, (B) Megavirus guangtanense, and (C) Moumouvirus qiuqiense, depicting the viral factory containing mature viral particles at 9 h p.i. (hours post-infection) and the stargate portal (black arrowhead) in the cytoplasm of infected *A. castellanii* cells.

**Fig. S2.** Biological properties of four new megamimivirus isolates. Histograms displaying the percentage of infectious viruses after treatments with different concentrations of (A) chloroform, (B) NaCl, (C) PBS at varying pH levels, (D) PBS under UV light after different exposure times, and (E) PBS at different temperatures. Data points represent mean values ± standard deviations from triplicate experiments. Asterisks denote statistical significance using the unpaired t test: **** indicate P-value lower than 0.0001.

**Fig. S3.** Coverage plots of (A) Megavirus honghaiwanense, (C) Megavirus guangtanense, and (E) Moumouvirus qiuqiense genomes by mapping clean reads from different sequencing platforms against the complete genome maps. Synteny analysis of the left terminal region (x-axis) of (B) Megavirus honghaiwanense, (D) Megavirus guangtanense, and (F) Moumouvirus qiuqiense complete genome maps compared with the right one (y-axis), highlighting the detailed comparison of the perfectly matched region.

**Fig. S4.** Coverage plots of Megavirus daqingense genome by mapping Illumina reads against the genome draft map using (A) Velvet, (C) A5-miseq, and (E) ABYSS assembly software. Synteny analysis of the left terminal region (x-axis) of (B) Velvet-based, (D) A5-miseq-based, and (F) ABYSS-based draft maps, with the right one (y-axis) highlighting the detailed comparison of the perfectly matched region. Synteny analysis of the left TIR (x-axis) of the Megavirus daqingense genome complete map with the partial (G) left or (H) right terminal regions (y-axis) of the ABYSS-based draft map.

**Fig. S5.** Synteny analysis of (A) *Mimivirus lagoaense*, (B) Mimivirus mama, (C) Mimivirus reunion, (D) Mimivirus terra2, (E) Mimivirus hirudo, (F) Mimivirus oyster, (G) Mimivirus bombay, (H) Mimivirus kasaii, (I) Mimivirus samba, (J) Mimivirus shirakomae, (K) *Mimivirus bradfordmassiliense*, (L) Mimivirus niemeyer of the left terminal region of draft map (x-axis) with right one (y-axis) highlighting the detailed comparison of perfect match region.

**Fig. S6.** Synteny analysis of (A) *Moumouvirus goulettemassiliense*, (B) *Moumouvirus moumou*, (C) Moumouvirus saudi, (D) *Moumouvirus australiense*, (E) *Moumouvirus lavasanguinense*, (F) Moumouvirus borely, and (G) *Moumouvirus maliense* of the left terminal region of draft map (x-axis) with right one (y-axis), highlighting the detailed comparison of the perfectly match region.

**Fig. S7.** Synteny analysis of (A) *Megavirus caiporense*, (B) *Megavirus powaiense*, (C) *Megavirus baoshanense*, (D) Megavirus avenue9, (E) Megavirus bus, (F) Megavirus T1, (G) Megavirus mammoth, (H) Megavirus T4, (I) Megavirus battle43, (J) Megavirus terra1, (K) Megavirus vitis, (L) Megavirus lba, (M) Megavirus courdo11, (N) Megavirus montpellier3, (O) *Megavirus chilense*, (P) Megavirus golden, (Q) Megavirus gilmour, (R) Megavirus shan, (S) *Cotonvirus japonicum*, (T) *Tupanvirus altamarinense*, (U) *Tupanvirus salinum* of the left terminal region of draft map (x-axis) with right one (y-axis), highlighting the detailed comparison of the perfectly match region.

**Fig. S8.** Comparison of (A) sequence similarity and (B) percentage of aligned sequence across the whole-genome, TIRs, and non-TIR regions (the genomic regions excluding the TIRs) between different isolates within the subfamily or the same genus. Asterisks denote significant differences using the unpaired t test: *, **, and **** indicate P-value lower than 0.05, 0.01, and 0.0001, respectively; “ns” indicates P-value > 0.05 (no significant difference).

**Fig. S9.** Partial-genome alignment of megamimivirus isolates. Mauve software was used with default parameters to align the non-TIR regions, in which the TIRs were removed from the whole genomes. Each genome’s panel contains a scale along the top axis showing the sequence coordinates for that genome, a single black horizontal center line to differentiate the forward and the reverse complement orientations, and the name of the genome sequence. Conserved genomic regions are indicated by colored locally collinear blocks, with identical colors across isolates representing homologous sequences.

**Fig. S10.** TIR alignment of (A) mimivirus, (B) moumouvirus, and (C) megavirus. Mauve software was used as described previously to align the TIR regions.

**Fig. S11.** Accumulation curves for (A) pan- and core- genomes and (B) pan- and core-TIRs. The pan-genomes/TIRs (teal boxes) represent the total repertoire of orthologous protein gene clusters, which increases with the addition of new megamimiviruses. The core-genomes/TIRs (purple boxes) represent the shared protein gene clusters conserved across all genomes, and their numbers decrease as the number of genomes increases. Box plots at each point display the median, interquartile range, and outliers resulting from random sampling of genome addition orders.

**Fig. S12.** Map of megamimivirus TIRs displaying the relative size, direction, and position of ORFs encoded in TIRs and non-TIR regions. ORFs in non-TIR regions are orthologous to (A) partial ORFs in TIRs, with the transposases highlighted (B). Links in different colors represent homologous proteins across different viruses.

**Fig. S13.** Distribution of (A-D) nonsynonymous substitutions value (dN) and (E-H) synonymous substitutions value (dS) across linear genomes of (A, E) Megavirus daqingense, (B, F) Megavirus honghaiwanense, (C, G) Megavirus guangtanense versus *Megavirus baoshanense*, (D, H) Moumouvirus qiuqiense versus *Moumouvirus australiense*. The x-axis represented the locations of genes on the linear genome. Asterisks denote statistical significance using the two-tailed Mann–Whitney test: *, **, ***, and **** indicate P-value lower than 0.05, 0.01, 0.001, and 0.0001, respectively.

**Fig. S14.** (A) Presence of identical Cys-tRNA, His-tRNA, and Trp-tRNA genes across different megamimiviruses. Numbers in blue cells represent the copy number of tRNAs in published genomes of each virus. The plot represents the presence or absence of Cys/His/Trp-tRNA genes, with red rectangles indicating those located within TIRs. The x‑axis labels correspond to representative Cys/His/Trp‑tRNA gene names, for which detailed information is provided in **Fig. S16**. Variation across (B) Cys-tRNA, (C) His-tRNA, and (D) Trp-tRNA gene regions in megamimiviruses. The x-axis is the DNA polymorphisms of tRNAs calculated by DnaSP (1 bp moving window average), and the y-axis is the position in the tRNA gene regions. Regions in the tRNA gene are color-coded according to secondary structure.

**Fig. S15.** Nucleotide sequence alignment of identical orthologue Leu-tRNA genes, which are flanked by 5’ and 3’ non-coding regions (A) within the genus *Megavirus*, and (B) across genera *Moumouvirus* and *Megavirus*. The information of tRNA gene positions is shown in the leaf names of phylogenetic trees in **Figure S15**.

**Fig. S16.** Maximum likelihood phylogenetic tree of (A) Leu-tRNA, (B) Cys-tRNA, (C) His-tRNA, and (D) Trp-tRNA genes based on nucleotide sequences using the *Fadolivirus algeromassiliense* as an outgroup. Numbers on each branch represent the percentage of trees supporting that branch (bootstrap values). Background colors of megamimivirus names represent different genera. The information of tRNA gene positions is shown in the first round-brackets of leaf names with anticodons in the second round-brackets.

**Fig. S17.** Nucleotide sequence alignment of paralogue Leu-tRNA genes encoded by (A) Megavirus daqingense, (B) Megavirus honghaiwanense, (C) Megavirus guangtanense, and (D) Moumouvirus qiuqiense. The information on tRNA gene positions is shown in the leaf names of phylogenetic trees in **Figure S15**.

**Table S1.** Geographic location of origin of the environmental samples.

| Virus | Sampling location | Sampling date | Geographic location | Sampling environment | Depth (m) |
| --- | --- | --- | --- | --- | --- |
| Megavirus daqingense | 44°49’’25’’ N, 129°46’28’’ E | Nov-17 | Heilongjiang, China | Production water, Daqing oilfield | 870~ 960 |
| Megavirus guangtanense | 23°55’8’’ N, 117°25’13’’ E | 23-Jan-24 | Fujian, China | Sediment, Zhangjiang mangrove reservior | 0 |
| Moumouvirus qiuqiense | 23°55’8’’ N, 117°25’13’’ E | 19-Jan-24 | Fujian, China | Sediment, Zhangjiang mangrove reservior | 0 |
| Megavirus honghaiwanense | 22°39’36’’ N,115°34’11’’ E | 23-Jan-24 | Guangdong, China | Saltwater, Red Bay | 0.5 |

**Table S2.** Statistics on the genome assemblies.

| Sequence platform | Marseillevirus | Megavirus daqingense | Megavirus honghaiwanense | Megavirus guangtanense | Moumouvirus qiuqiense |
| --- | --- | --- | --- | --- | --- |
| Illumina | Number of clean reads | 11,590,522*2 | 44,413,773*2 | 28,755,069*2 | 9,533,715*2 |
|  | Total clean base / nt | 3,493,752,682 | 13,291,678,544 | 8,640,020,754 | 5,720,241,859 |
|  | Average size / bp | 151 | 149 | 150 | 150 |
|  | Average depth/ % | 769 | 7,919 | 6,271 | 2,356 |
| Nanopore | Number of clean reads | 88,461 | 184,313 | 339,718 | 194,859 |
|  | Total base / nt | 1,269,502,139 | 1,096,460,093 | 1,908,493,587 | 999,211,720 |
|  | Average size / bp | 7,577 | 5,948 | 5,617 | 5,127 |
|  | Average depth/ % | 710 | 683 | 808 | 1,424 |
| PacBio | Number of clean reads | 3,447,912 | 2,475 | 6,668 | 7,278 |
|  | Total base / nt | 682,551,799 | 22,388,753 | 59,661,917 | 69,663,674 |
|  | Average size / bp | 7,715 | 9,005 | 8,947 | 9,571 |
|  | Average depth/ % | 479 | 17 | 48 | 65 |
| Total length / bp | | 1,265,278 | 1,248,390 | 1,228,839 | 1,067,849 |
| GC content / % | | 25.48 | 25.29 | 25.35 | 25.22 |
| Number of CDS | | 1,192 | 1,158 | 1,171 | 1,032 |
| Intergenic distance / nt | | -225 ~ +4,327 | -70 ~ +1072 | -69 ~ +1627 | -85 ~ +1120 |
| Coding density / % | | 68.23 | 90.33 | 90.20 | 90.58 |
| Aminoacyl-tRNA synthetase (aaRS) | | TyrRS, TrpRS, CysRS, ArgRS, MetRS, AspRS, AsnRS (2×), IleRS (3×) | TyrRS, TrpRS, CysRS, ArgRS, MetRS, AsnRS, IleRS | TrpRS, CysRS, ArgRS, MetRS, AsnRS, AspRS, IleRS | CysRS, MetRS, AsnRS |
| tRNA | | Leu (4×), His, Cys, Trp | Leu (4×), His, Cys, Trp | Leu (4×), His, Cys, Trp | Leu (4×), His, Cys |
| Translation factors | | IF4A, IF4E, SUI1, GTPBP, eRF1 | IF4A, IF4E, SUI1, GTPBP, eRF1 | IF4A, SUI1, GTPBP,  eRF1 | IF4E, SUI1 |
| GenBank Accession | | MT663335.2 | PQ010613.1 | PX691300.1 | PX691301.1 |

**Table S3.** Megamimivirus genomes used in this study.

| *Megamimivirinae* | GenBank accession number | *Megamimivirinae* | GenBank accession number |
| --- | --- | --- | --- |
| *Mimivirus bradfordmassiliense* | HQ336222.2 | Megavirus T1 | LN868526.1 |
| *Mimivirus lagoaense* | KM982402.1 | Megavirus T4 | LN869537.1 |
| Mimivirus mama | JF801956.1 | Megavirus avenue9 | LN867403.1 |
| Mimivirus bombay | KU761889.1 | Megavirus bandra | MG779341.1 |
| Mimivirus hirudo | KF493731.1 | *Megavirus chilense* | JN258408.2 |
| Mimivirus kasaii | AP017644.1 | *Megavirus baoshanense* | MH046811.2 |
| Mimivirus niemeyer | KT599914.1 | *Megavirus powaiense* | KU877344.1 |
| Mimivirus oyster | KM982401.1 | Megavirus battle43 | LN868256.1 |
| Mimivirus reunion | MW004169.1 | Megavirus bus | LN868539.1 |
| Mimivirus samba | KF959826.2 | *Megavirus caiporense* | OP925046.1 |
| Mimivirus shirakomae | AP017645.1 | Megavirus courdo11 | JX975216.1 |
| Mimivirus terra2 | NC_023639.1 | Megavirus courdo7 | JN885991.1 |
| *Moumouvirus moumou* | JX962719.1 | Megavirus gilmour | MG602507.1 |
| *Moumouvirus australiense* | MG807320.1 | Megavirus golden | MG602508.1 |
| *Moumouvirus goulettemassiliense* | KC008572.1 | Megavirus lba | JX885207.1 |
| Moumouvirus borely | MN175499.1 | Megavirus mammoth | OQ411602.1 |
| *Moumouvirus lavasanguinense* | LC813553.1 | Megavirus montpellier3 | LN868518.1 |
| *Moumouvirus maliense* | MK978772.1 | Megavirus shan | LN868520.1 |
| Moumouvirus saudi | KY110734.1 | Megavirus terra1 | KF527229.1 |
| *Cotonvirus japonicum* | AP024483.1 | Megavirus vitis | MG807319.1 |
| *Tupanvirus salinum* | KY523104.2 | *Fadolivirus algeromassiliense* | MT418680.1 |
| *Tupanvirus altamarinense* | MF405918.2 |  |  |
